# Perspectives on Cognitive Therapy Training within Community Mental Health Settings: Implications for Clinician Satisfaction and Skill Development

**DOI:** 10.1155/2012/391084

**Published:** 2012-09-29

**Authors:** Shannon Wiltsey Stirman, Christopher J. Miller, Katherine Toder, Amber Calloway, Aaron T. Beck, Arthur C. Evans, Paul Crits-Christoph

**Affiliations:** ^1^VA National Center for PTSD, VA Boston Healthcare System, Boston University, Washington, DC 20420, USA; ^2^Center for Organization, Leadership, and Management Research, VA Boston Healthcare System, Washington, DC 20420, USA; ^3^Department of Psychiatry, University of Pennsylvania, Philadelphia, PA 19104, USA; ^4^Department of Behavioral Health and Intellectual disAbilities Services, Philadelphia, PA 19107, USA

## Abstract

Despite the mounting evidence of the benefits of cognitive therapy for depression and suicidal behaviors over usual care, like other evidence-based psychosocial treatments (EBTs), it has not been widely adopted in clinical practice. Studies have shown that training followed by intensive consultation is needed to prepare providers to an appropriate level of competency in complex, multisession treatment packages such as cognitive therapy. Given the critical role of training in EBT implementation, more information on factors associated with the success and challenges of training programs is needed. To identify potential reasons for variation in training outcomes across ten agencies in a large, urban community mental health system, we explored program evaluation data and examined provider, consultant, and training program administrator perspectives through follow-up interviews. Perceptions of cognitive therapy, contextual factors, and reactions to feedback on audio recordings emerged as broad categories of themes identified from interviews. These factors may interact and impact clinician efforts to learn cognitive therapy and deliver it skillfully in their practice. The findings highlight experiences and stakeholder perspectives that may contribute to more or less successful training outcomes.

## 1. Introduction

The public health impact and high rates of depression and suicide in community populations are well established [1–5]. Accumulating evidence indicates that providing psychosocial evidence-based treatments (EBTs) such as cognitive therapy (CT; [6]) results in substantial benefits to physical and mental health symptoms, quality of life outcomes, and reduction of health disparities and suicide attempts [7–9]. Training clinicians in community mental health agencies to provide CT for depression can result in improved treatment outcomes [8]. Like other EBTs, however, CT has not been widely adopted in clinical practice. Until recently, few systematic implementation efforts or training opportunities existed in the public sector [10, 11]. In response to the shortage of adequately trained providers [10], policymakers have devoted substantial resources in recent years to train providers in EBTs in the context of large-scale implementation programs [11, 12]. 

In 2007, the Beck Initiative was formed as a collaborative partnership between the Philadelphia Department of Behavioral Health and Intellectual disAbilities Services (DBHIDS), and the University of Pennsylvania (Penn) to implement CT within the city's behavioral health provider agencies. The mental health system administrators became interested in implementing CT because it has a strong evidence base for multiple disorders and client populations, it can prevent relapse and reduce costs associated with treatment, and it has been shown to improve quality of life [13–15]. In determining the disorders to target in the first phase of the training program, the partnership members chose depression and suicidal behavior for three reasons. First, a high proportion of consumers in the system have a diagnosis of depression. Second, they believed that training in CT basics as applied to depression would serve as a good foundation for more specialized trainings. Third, because suicide prevention is a high priority, after the results of a recent clinical trial demonstrated significant benefits of CT over and above usual care for highly comorbid suicidal individuals in the community [9], partnership members were interested in rapidly making the treatment available throughout the system. Because psychosocial treatments for depression tend to be complex multisession treatment packages [16, 17], research has demonstrated that one-time trainings without support in the form of expert consultation are insufficient to promote skill development and behavior change [18–24]. Thus, both partners were committed to following up training workshops with an intensive consultation component that included weekly discussion of cases combined with expert review of, and feedback on, clinician's efforts to provide cognitive therapy to their clientele.

Beyond the growing evidence regarding the importance of consultation in EBT implementation, limited information is available about determinants of success, or shortcomings of training and consultation strategies. Research to date has focused on comparisons of front-end training strategies and examinations of the impact of adding consultation. Little research has been conducted to facilitate understanding of the key processes in training and consultation. Qualitative research with stakeholders can complement the existing empirical literature by providing valuable insight into aspects of training and consultation programs that may impact the success of implementation efforts. 

The purpose of this study is to identify aspects of the training and consultation process that may be associated with CT skill development and clinician satisfaction with training. To do so, we explored program evaluation data and provider, consultant, and training program administrator perspectives on training and consultation that occurred in the context of a community-academic partnership to implement CT. In contrast with published randomized controlled trials of training and consultation, which have included individually recruited clinicians, this study examined training that occurred with cohorts within ten agencies in an urban community mental health system. 

## 2. Method

### 2.1. Training Program

#### 2.1.1. Setting

 DBHIDS is a large mental health system with over 300 provider agencies that are heterogeneous in size, structure, populations served, and availability of resources. These agencies provide care to serve the mental health and substance abuse needs of the city's 420,000 Medicaid recipients. Recent utilization data indicate that approximately 38% of the clients in the mental health system have a diagnosis of major depressive disorder (28%) or depression NOS (10%), and 87% of these clients receive psychotherapy [25]. 

#### 2.1.2. Treatment

 CT is a psychosocial treatment that identifies and changes dysfunctional patterns of thinking, behavior, and emotional responses by helping individuals develop skills for modifying beliefs, relating to others in different ways, and changing behaviors through the use of a variety of interventions [14]. The structure of the session is intended to reflect the active, goal-oriented, collaborative nature of the treatment. Sessions are structured to include a brief summary or bridge between the previous and current session; efforts to set and follow an agenda; review of practice, or “homework” completed between sessions; summaries of important points; feedback from clients on their experience and understanding of the concepts covered in the session and efforts to identify a way to practice new skills between sessions [26]. Competence at structuring sessions as measured by the structure subscale of the Cognitive Therapy Rating Scale (CTRS; [27, 28]), and the assignment and completion of homework [29, 30], have been shown to predict greater symptom reduction in CT. 

#### 2.1.3. Training and Consultation

 The training program was designed to teach CT basics, with a particular emphasis on depression and suicidal behaviors. It included extensive guidance on using CT to address commonly cooccurring problems among individuals who present with symptoms of depression. Training consisted of 24 hours of workshops followed by six months of weekly consultation [31]. The program was administered by a project director at Penn and by a full-time operations specialist within DBHIDS. To address financial constraints to participation in training, agencies were reimbursed for time spent in training and consultation activities. A clear expectation of the program was that agencies continue to support the ongoing use of CT after training: clinicians were trained in cohorts within their agency so they could build a community of practice, and throughout the program, the partnership worked with agency leadership to facilitate support [31, 32]. The approach to training was based on information obtained through a needs assessment [33] and a review of literature from a variety of disciplines [34]. Given the evidence that a multilevel approach is critical to implementation, the training program was embedded within a broader program to implement EBTs within the system [31, 32]. External facilitation, a process of interactive problem solving and support [35] that has been shown to be effective in engaging stakeholders, addressing potential barriers to implementation, and increasing the use of EBTs [36–39], was integrated into the training program. 

The training model that was developed, the ACCESS training and consultation model, included the following components [32]: *Assess *needs and barriers (engage stakeholders and assess through preliminary meetings, surveys, work samples, interviews) and* Adapt* training content and materials as required, *Convey the basics* through initial didactics, *Consult* on case material and on strategies to overcome barriers during consultation and through periodic meetings with key personnel, *Evaluate work samples *to provide feedback and refine skills, *Study outcomes *in ways that are feasible and acceptable to the agency, and *Sustain *by anticipating and addressing future barriers, maintaining communication, and making a plan for training future staff. The evaluation of work samples occurred in two formats. Initially, Penn consultants reviewed audio recordings of sessions and provided feedback through telephone consultation with individual clinicians, and a group consultation meeting was held to discuss more general CT skills. Later, in response to concerns about the time, costs, and scalability of this approach, individual consultation was replaced with review and discussion of segments of session recordings in a group consultation format. Additionally, as the goal of the program was to promote ongoing CT skill development and fidelity after training, the group feedback format was viewed as preparation for the groups to transition to ongoing peer-led group session review. During the consultation phase, consultants also provided individualized feedback on one to two full sessions for each participant in the group consultation model. Two clinicians who struggled to master CT in this model were given additional feedback and consultation. 

#### 2.1.4. Selection of Participating Agencies and Clinicians

Agencies were nominated for participation in the training program by mental health system administrators, who attempted to make the training available to a variety of programs serving diverse populations throughout the city. The training director at the university made a final selection for each round of training after reviewing information about the agency and the populations that it served and taking into consideration the areas of expertise of available consultants. Key personnel meetings were held with agency administrators to discuss the program, assess fit, and build collaboration. Agencies had latitude to select clinicians for participation internally, although criteria for inclusion set forth by the training program were that participants should have an interest in learning CT and be willing and able to participate fully in the program (e.g., record sessions with training cases and attend training workshops and consultation meetings). Consultation was limited to eight or fewer participants at each agency, regardless of agency size, although some agencies sent additional employees or supervisors to the initial workshop. Participants completed an application that included information about their prior training and reasons for wanting to learn CT. Applications were used primarily to understand clinicians' backgrounds prior to training, and no clinicians who were selected by their agencies were denied participation in the training program. To assess and facilitate engagement, prior to the workshops, preliminary meetings were held with the clinicians (with no management present) to describe the training program, emphasize the voluntary nature of the program, and discuss any questions or concerns that clinicians expressed. Training generally occurred in groups of providers from a single agency, but on two occasions, trainings included groups of providers from two agencies. A total of 99 providers of adult outpatient services at ten agencies enrolled in the basic training program.

#### 2.1.5. Criteria for Successful Completion of Training

 Providers were eligible for designation as skilled providers of CT (hereafter described as “passing” for brevity's sake) if they attended at least 80% of the weekly consultation sessions over 6 months, submitted at least 20 recordings of sessions with clients who agreed to serve as training cases, and achieved a rating of at least 40 on the CTRS, a validated measure of competence in CT [40]. A score of 40 was chosen as the passing score because it is the standard minimum required competence score for clinicians who serve as therapists in clinical trials of CT for depression [41].

### 2.2. Study Procedures

The study was approved by the Penn and City of Philadelphia Institutional Review Boards. All of the clinicians who participated in one of twelve training programs at ten agencies were invited to participate in interviews regarding their use of CT in adult outpatient service settings. Forty clinicians expressed interest in participating, but after multiple attempts, twenty-six clinicians were able to be scheduled for posttraining interviews. Clinicians who participated in interviews consented to allow their CTRS scores to be linked to their interview data. Training consultants (*n* = 6) and program administrators from the university (*n* = 3) who were involved in the program were also interviewed. To ensure that our sample would reflect a broad range of perspectives, we also evaluated anonymous feedback forms (free response and numeric ratings) from training and consultation that were collected as program evaluation data.

Participants were scheduled for interviews at times that were convenient for them and that would not interfere with their clinical and work-related responsibilities. Semistructured interviews were conducted over the telephone or in person and interviews were digitally recorded. An interview guide included open-ended, scripted questions that were based on the Promoting Action on Research Implementation in Health Services (PARIHS) Framework [42]. Participants were given the opportunity to elaborate on issues that they considered particularly relevant or about which they expressed strong sentiments. Interviews typically lasted 45 minutes to one hour. Participants received financial compensation or a gift card for their participation. Interviews were transcribed and transcripts were reviewed and checked for accuracy by at least one of the authors. 

### 2.3. Coding Procedures

Clinician interviews included questions about a variety of factors, but for the current paper, we will focus on responses that address training and consultation. Transcripts of interviews and program evaluation data were subjected to qualitative analysis rooted in grounded theory [43], using the ATLAS.ti software package [44]. First, study investigators developed a list of a priori themes and emergent themes were added after reading the interview script and several transcripts. After saturation was achieved (i.e., the coding of additional transcripts did not identify any further themes to be coded), a preliminary codebook was developed based on the identified themes. This preliminary codebook was then used by all of the study researchers to rate two additional transcripts, resulting in discussion and revisions to the codebook based on group consensus. Using this revised codebook, two study researchers rated the remaining transcripts. A subsample of transcripts was coded by both raters, and disagreements regarding coding were resolved via consensus of the study team in weekly coding meetings. This process also identified several additional subcategories of codes. A process of axial coding suggested ways in which some codes could be combined or collapsed into one another, and arranged all of the codes into a treelike, hierarchical structure. The result of this process was a finalized codebook, with links specified between key concepts. The two primary raters reviewed transcripts and updated their ratings to reflect the themes from this finalized codebook. Program evaluation data was integrated with interview and free response data, and through a process of triangulation and constant comparison, the categories were organized around the major emergent themes. 

## 3. Results 

### 3.1. Participant Characteristics

 The 26 clinicians who participated in interviews were 66% female and had an average of 5.7 years of experience working in mental health treatment settings (sd = 1.3). Eighty percent had a Master's degree, 7% had completed some graduate work (e.g., towards a Master's Degree), and 13% had a Bachelor's degree. Participants were 70% Caucasian, 19% Black, 4% Asian, and 7% were multiracial or endorsed a different race or ethnicity. Seven percent of the participants were Latino. Participating agencies varied in size, with anywhere from 5 to over 50 clinicians serving their adult outpatient clientele. Most agencies provided general outpatient mental health services, but four agencies, or subprograms within the agencies, that served specific populations participated in the training. These populations included individuals with severe mental illness, specific minority populations, and individuals in recovery from substance use disorders. Additionally, one agency included clinicians from their specialized program for severe mental illness as well as from their general outpatient mental health program. 

### 3.2. Training Program Outcomes

Table 1 provides an overview of the proportion of clinicians who passed at each agency, the feedback and consultation format used, information about whether the agency specialized in treatment for specific client populations or diagnoses, and information on retention in the program. Overall, approximately 80% of the clinicians who completed consultation passed. At four agencies, one or two clinicians did not complete consultation. Program evaluation data revealed that attrition was due to relocation, layoffs due to budget constraints, medical leave, or taking a new position. At two agencies, approximately half of their clinicians passed the trainings; the other agencies had higher pass rates. One of the agencies with lower pass rates provided specialized services and the other provided general mental health services. Of the 26 clinicians who participated in interviews, seven clinicians from five different agencies did not pass. Due to the sensitive nature of some of the information that participants disclosed, and to ensure that agencies could not be identified, we have not revealed exact numbers of training participants or clinicians who passed the training, or linked quotations to agency IDs or more specific information about the agencies. 

Program evaluation data indicated that overall satisfaction with the program was generally high. The mean overall quality rating for the individual feedback model was 4.88 (SD = 1.4) on a 6-point Likert scale, and the group feedback model was rated 5.63 (SD = .52). After training, clinicians who participated in the individual model rated their comfort in applying CT to be a mean of 3.81 (SD = 1.5) and participants in the group format expressed a mean comfort score of 4.88 (SD = .64). 

### 3.3. Themes Related to Training Success

As described above, an axial coding process was used to identify major themes from the qualitative interview data. Five themes relevant to training success emerged during the coding process, which were grouped into three broad categories: perceptions of CT (relevance to agency clientele and perceptions of CT structure); contextual factors (agency involvement and impact of clinician selection process); and experience of consultation and feedback on recordings. 

#### 3.3.1. Perceptions of Cognitive Therapy

**Relevance of CT to Agency Clientele**

In light of pre-training findings that some clinicians within the system had doubts that CT could meet the needs of some of their clients [33], efforts were made to match consultants who had clinical experience with similar populations and to identify appropriate video and case examples. All five consultants described efforts to use relevant case material and demonstrate ways in which case conceptualization could facilitate the appropriate application of CT strategies to common cooccurring presenting problems. Interview data indicated that this strategy was somewhat successful. Most clinicians expressed openness to the use of CT with their clientele. At an agency that provided general mental health services, a clinician stated the following.

*Clinician: We watched tapes of lots of the cognitive therapists conducting sessions with clients that were more similar to the ones we dealt with. Certainly I could see where that was helpful, where it worked… I feel that it could work absolutely with our population… but only if the therapists were as invested in the technique.*

Clinicians in some agencies also expressed appreciation that consultants appeared to have experience in working with clients like theirs. In contrast, at an agency where fewer clinicians passed, a consultant for that agency described challenges in addressing concerns about the use of CT with the clientele at that agency.

*Consultant: Training was very difficult at that agency because from the beginning we had low buy-in from the staff and they had difficulty seeing the application of CT for their clients and they really struggled with taping, with utilizing CT techniques.… (The other consultant) and myself tried to discuss clients that we had seen with very similar levels of functioning…. it was kind of general community mental health depression, kind of the multi-disordered. And then also some people with psychotic disorders.… I do not see that population being any different from the other agencies. *

However, a clinician at that agency expressed a very different perspective, “my biggest struggle was with [the consultants], [who] really never gave ample time or conversation to my population and that might be because they never had any experience in it.” At another agency where fewer clinicians passed, clinicians expressed skepticism that CT was presented as applicable to a variety of presenting problems and felt that consultants were not open to their efforts to discuss the limitations of CT. 

Interviews with three of the consultants and two training program administrators suggested that the strategy of teaching CT basics as applied to depression and suicide may have generated some impatience among clinicians. Many expressed interest in guidance on addressing issues that they perceived to be more difficult to address in treatment, such as psychosis, Axis II diagnoses, and active substance dependence. A consultant described this concern at an agency that offered specialized services.

*Consultant: Although they really were open and interested and invested in learning, I think they struggled with the fit and they were sort of assured along the way, “let us get the basics down and then we will think about how to apply it to this very challenging population.” But they struggled I think with that, to the extent that the training program rethought that approach to some degree.*

**Reactions to CT Structure during Training**

Despite previously documented concerns within the system about the potential for CT to be rigid [33], most clinicians did not express strong concerns about its structure. One clinician found that the training disabused her of stereotypes in this domain: “… it's really supportive, and it's pretty flexible, so I think the training was good to break that stereotype that CT is going to be very structured and rigid [without] a lot of room for other opportunities to connect with people.” Some clinicians found the structure to be beneficial for clients, with one indicating on the program evaluation survey, “Agenda setting has helped me organize the sessions and helped my clients focus and create their own solutions to their problems, which is very efficient and respectful.” Additionally, some noted the positive impact of using the CT structure on with clients who presented as disorganized or “scattered.”

Some negative reactions to CT structure were described, however, particularly at agencies where fewer clinicians passed. Consultants identified challenges related to providing feedback to clinicians who demonstrated ongoing discomfort with the structure of CT. A consultant explained that structuring sessions was challenging for some clinicians.

*Clinician: I do not think that she was as comfortable with the structure or with delivering the therapy. It took a fair amount of work to get her to really start delivering the intervention with the training cases that she had.… it was kind of a hard sell for her to implement it. *

Without the structure in place, consultants stated that sessions “did not look like CT sessions” and CT interventions were delivered sporadically or unsystematically. Consultants described efforts to balance this feedback regarding structure with their ongoing efforts to simultaneously emphasize case conceptualization and selection of appropriate interventions.

*Consultant: I did not want anything that I did to turn them off to [CT] so I tried to hear out their concerns…. but then [it was also important to] make sure that they got what they needed to really be able to deliver the treatment….*

However, at the two agencies with lower pass rates, clinicians expressed concern that training overemphasized the structure of CT, to the detriment of patient-centeredness; as one clinician concluded, “it seemed that the structure was more important than helping the client.” 

CT structure was initially perceived by some clinicians who ultimately passed to be intimidating or challenging to master.

*Clinicians: I think when I started the training I was kind of afraid of all of it because… it was so much for me… in terms of me thinking about the structure and knowing where I was, and pacing, and summarizing, and mood check. But now that I do it fairly regularly, I think it's okay.*

Perhaps due to the perception that the CT structure was challenging to implement, some clinicians who maintained structured sessions during the training indicated that they “loosened up” the structure soon after they completed training.

*Clinician: When I would have a consultation session I was doing pure CT. And when I know that no one's going to be supervising me—listening, and so forth—I do not feel that pressure. I feel like I can relax a little more. And relaxed to me means it's okay if I do not hit all the points. *

#### 3.3.2. Contextual Factors

**Administration and Supervisor Involvement**

Although the training model included initial and periodic meetings with agency administration and supervisors to assess needs, obtain feedback, and discuss progress, clinicians and consultants perceived varying levels of day-to-day involvement and enthusiasm. For example, at an agency at which most clinicians passed, a consultant noted the following.

*Consultant: [Clinic leaders] were really supportive and excited, and the head of the agency was very involved in preliminary meetings and was definitely present. She went to all of the check-in meetings and then they put a fairly high level administrator and supervisor in the training… so that [they] could support CT within the agency.*

In contrast, at an agency at which fewer clinicians passed, a consultant noted, “we were concerned about how things were unfolding and [the administration] seemed not particularly concerned… it was problematic for the training. Without agency buy in, we're asking a lot of these therapists*…*.” Administration at agencies where more clinicians passed set aside protected time for CT training, invited board members and other administrators to celebrations to express appreciation to the participants in the program, effectively engaged in problem solving, and continued to allocate time for CT-trained clinicians to meet for ongoing peer consultation after training was completed. 

In contrast, at three agencies, two of which had lower passing rates, upper level management voiced, and at times demonstrated, support for the training program, but mid-level management appeared to be less invested. At one agency with low clinician engagement and low CTRS scores at the midpoint of the training, consultants attempted to discuss progress, get feedback, and problem solve with the clinicians. When this was not effective, they attempted to work with supervisors and management to improve the training process and increase engagement. At one agency, this process appeared to backfire.

*Consultant: [The supervisors] went back and said [to the clinicians], “well the consultants said you aren't doing this right so fix it.” And the result of that was that the trainees got really frustrated with us and really angry with us because they thought that we went over their heads.*

The consultant described the upper management's subsequent efforts to try to support the process, but “We did not see much result from that. Maybe a little bit more participation for a week or two but then it kind of died down.”

**Perceived Pressure or Mandate to Participate in Training**

A salient issue raised by clinicians and consultants at three agencies was a mandate, or a perception of a requirement, by their agency to participate in the training program. One clinician felt uncomfortable with the feeling of pressure to participate, but nonetheless found the training helpful.

*Consultant: Had I felt free to decline, that would have been more helpful because I would have [declined the training], not because I did not appreciate the training, but because I felt that it could have been somebody else who might have utilized it. *

In contrast, the mandate to attend the training was particularly onerous for clinicians whose initial attitudes toward CT were unfavorable.

*Clinician: We were not told, “Hey, do you want to do this thing? Do you want to learn about CBT?” We were told, “You are going to this.” So, it was mandatory, so I really wasn't on board with that because I did not really have an interest in learning about it. *

The apparent mandate from some agencies to complete CT trainings was also noted by the consultants. For example, one consultant described it as a “major issue” for one agency and then described a consultation meeting at which it was discussed.

*Consultant: That was the top [concern] on their list-was that they had been required, mandated by their agency to do this and that that had sort of set the wrong tone for them, and I had a lot of conversations with them about our intentions … that it was important that this be voluntary. And they said that if we went back to their agency and said that they had admitted that they had been mandated to do it that they were afraid that they'd lose their jobs. And so that really ties our hands… it set the tone in sort of a negative way to be forced to do this. *

After this experience, individual interviews with clinicians were added to assess their interest in the program and their training needs. Despite these steps, clinicians appeared to perceive pressure to participate at an agency that subsequently participated in the program. As a consultant stated “We did do the interviews prior to starting … And I remember that I personally did the interview for the person that was the hardest and she swore up and down how much she loved CT.” Notably, at two of the three agencies at which clinicians described a perception of pressure to participate had lower passing rates. 

#### 3.3.3. Experience of Consultation and Feedback on Recordings

Consistent with program evaluation data on satisfaction, nearly every clinician stated that recording sessions were ultimately beneficial. Participants expressed appreciation for individual feedback, with one clinician stating that “The consultation with [consultant] was probably one of the best supervisions that I've ever experienced. I was able to learn things from her and take it and put it into action.” Many commented that they felt that the feedback, whether in group or individual format, was supportive and nonjudgmental. Advantages to group feedback that were noted by consultants and clinicians who participated in the group format included hearing how their colleagues applied CT to a variety of cases and supporting one another's challenges and success as they learned. Perhaps surprisingly, clinicians expressed little resistance to group review of their session recordings. Program evaluation data and interviews also indicated that more time spent in role plays during workshops and consultation would have been appreciated, indicating that clinicians were willing to engage in potentially uncomfortable training experiences if it could improve their skills. Despite apprehension about playing sessions for peers, clinicians' and consultants' anxiety decreased over time, and ultimately this supervision format increased their skills. As one clinician stated that “Peer and consultant feedback is crucial to my learning. While humbling and at times anxiety provoking, the feedback is always supportive and gives me great direction for ongoing learning, in addition to validating what I am doing well.” 

At an agency where fewer clinicians passed, though, some clinicians did not appear to perceive the group feedback model to be sufficient or supportive.

*Consultant: We were getting frustrated because we would play [examples of CT] that were clearly different from tapes [a clinician] would play. And she would say “I just did the same thing and you're saying that that's good and what I'm doing is not good.” And even when the other clinicians would try to point out the differences, she would just get really shut down and really annoyed to the point that I think the other clinicians were afraid to say anything to her. *

A therapist at the same agency stated that if more individual feedback on his or her own cases had been provided, the recordings would have felt more useful, “If you're not getting feedback on a regular basis and you're not hearing a whole session then you do not get the complete feedback that you need.” 

Some clinicians who did pass expressed a desire for ongoing feedback after training was complete, suggesting that this aspect of training was particularly valued. They indicated that they believed that the quality of the treatment they provided could be improved by ongoing recording and feedback after training was completed. One clinician, for example, stated that “I would say when I was getting regular supervision from [the consultant] and my sessions where being taped, they were working pretty well; now I think it's hit or miss at best to tell you the truth.” 

## 4. Discussion

This study reported on the experiences of clinicians, consultants, and administrators during a CT training and implementation program in an urban behavioral health system. Unlike previous studies, this study examined training that occurred with cohorts within agencies rather than individually recruited clinicians. This difference allowed us to investigate the ways in which organizational context may influence the experience of training. Our findings are illustrative of the PARIHS model, which suggests that successful implementation is a function of perceptions of the intervention and its effectiveness, context, and facilitation [42], and they suggest ways in which these three elements may interact and influence the process of training and consultation. Feedback and opportunities to discuss session recordings were identified as important facilitators of clinician skill and comfort in delivering CT, and administrative support appeared to play a role in success. At agencies with lower pass rates, however, pressures that may have been felt by some administrators (e.g., to encourage attendance at trainings), consultants (e.g., to facilitate CT skill development), and clinicians (e.g., to attend trainings despite misgivings about CT) may have led to patterns of interactions that reduced the likelihood of successful training outcomes. Perceived mandates and agency climate, combined with initial concerns about CT, may have set up a dynamic in which doubts about CT's structure and its relevance to a given population resulted in a greater struggle, or less desire, to utilize CT on the part of the clinicians. The resulting feedback, focusing on increasing the use of CT interventions and procedures, may have simply confirmed initial, negative perception of CT as structured and rigid, and set up a dynamic by which consultants and clinicians became increasingly discouraged with the training experience. Future study with a larger sample is necessary to investigate this hypothesis more systematically.

These findings provide a richer explanation of processes that may underlie previous findings that indicate that agency context and administrative support can influence clinician attitudes [45, 46] and the implementation of EBTs [47]. Supportive actions and the provision of resources by leaders (e.g., protected time for trainings and public acknowledgments of clinicians undergoing the training) were perceived as helpful. On the other hand, administrative mandates or pressure to enroll in training did not appear to be successful, and more research on the impact of mandates on clinician attitudes and experience of training is warranted [48]. However, our results do not suggest that training programs should rule out potential participants solely because they have initial doubts about the potential effectiveness of CT. Such doubts were expressed by some clinicians who willingly participated, experienced a change of opinion, and ultimately passed the training. Future research should investigate whether the addition of an assessment of psychological safety or organizational social context (c.f., [47]) prior to the selection of participating agencies can decrease the likelihood of pressure or compulsory participation. Addressing organizational context can be a sensitive and challenging issue, and allowing or encouraging uninterested clinicians to opt out of consultation requires sensitivity to the organizational climate, the potential impact on partnerships and relationships with agencies, and clinician morale. For agencies with more challenging organizational contexts, facilitation may be insufficient and more intensive organization-level interventions might be necessary to promote successful outcomes [49]. In other contexts, particularly after a training program matures and publicizes success, clinicians may be more likely to volunteer for training. Since the initial twelve trainings that were the focus of this report have occurred, more clinicians in the system have been approaching their agency administration with requests to participate in the training program.

Some of our findings may suggest the possibility that a less structured EBT or a more modular approach would be better received by some clinicians [50, 51]. However, some clinicians ultimately viewed session structure to be helpful for clients. Achieving a balance between emphasis on structure as a key element of CT and guidance on how to personalize treatment and increase clinician comfort appears to be crucial, yet challenging, particularly in light of concerns about the treatment that may be present prior to training [33, 52, 53]. The tendency of some clinicians to “loosen” the structure of the sessions after training was completed also warrants attention, as doing so may decrease the intervention's effectiveness and ultimately contribute to or reinforce perceptions that CT is not effective in community settings. Followup strategies such as ongoing consultation or fidelity monitoring may be effective methods of supporting sustained implementation [32, 54].

The results of this study highlight an important challenge in providing training in depression treatments in community behavioral health settings and systems. A significant proportion of individuals enrolled in publicly funded mental health systems are diagnosed with depression [25]. Yet the strategy of providing a general depression- and suicide-focused training as a foundation for future, more specialized trainings appeared to prove unsatisfying to some clinicians. Interviews with consultants and clinicians revealed that they perceived a need for training to address challenges related to severe mental illness, comorbidities, and diagnostically complicated clients. In response to stakeholder feedback on the training program, considerable effort was devoted to finding video clips of individuals who resembled the populations served by the agencies. Professionally produced video materials did not meet all of the needs expressed by training participants, but some videos of clients from clinical trials who agreed to allow their case material to be shared for training purposes were identified. However, to meet stakeholder needs, the training program has also expanded to include more specialized training programs for psychosis, substance dependence, and personality disorders. 

Overall, a majority of clinicians interviewed for this study reported having a good experience with the training program for depression and suicide. Most viewed the review of session recordings as a critical component of their training. This finding is particularly important because some large-scale EBT training programs do not include the use of session recordings [11]. Further investigation is warranted to determine whether this component improves training outcomes over and above consultation without review of work samples. Clinicians reported appreciation for feedback, including group-based feedback on recorded sessions, despite some initial discomfort with sharing their work in a group setting. While the mean rating for confidence in using CT appeared to be higher among clinicians who participated in group consultation sessions than for those in individual consultation, the sample size and nesting of clinicians within cohorts precluded an appropriate statistical comparison between groups. It is possible that there are some benefits to a group feedback model, such as cost efficiency, exposure to examples of the use of CT with a wider variety of cases, and supportive feedback that may increase confidence. Additionally, after expert consultation is complete, ongoing peer group consultation may help clinicians maintain their adherence to CT. However, as some clinicians noted, the tradeoff to this condition is less intensive individualized feedback. Future comparisons of the two consultation models in terms of costs, fidelity, and client outcomes would be useful in determining the most advantageous models.

Findings from this study should be interpreted in light of several limitations. Our interview sample was relatively small, and individuals with stronger or more extreme opinions may have been more likely to participate. We attempted to mitigate this limitation by coding anonymously provided program evaluation data as well. This study was not designed to test hypotheses, but rather to inform further investigation of potential determinants of successful training outcomes, and to reveal potential reasons for the variations in the numbers of clinicians who passed at different agencies. Despite these limitations, we identified several directions for future research. The relative benefits and disadvantages of providing training in evidence-based interventions to clinicians within their organizational setting should be further explored. It will be important to test strategies to address organizational climates that are less favorable for training and implementation. Additional research is needed to determine whether feedback on work samples enhances clinician skills over and above consultation alone. 

## Figures and Tables

**Table 1:** 

Agency ID	Consultation and feedback model	Attrition from consultation?^†^	Proportion of completers who passed	Offered specialized treatment for a specific population
A	Group	yes	70%	*✓*
B	Individual	0	83%	*✓*
C	Individual	0	100%	
D	Individual	0	50%	*✓*
E	Individual	0	100%	
F	Group	yes	70%	*✓*
G	Group	0	65%	*✓*
H	Group	yes	70%	
I	Group	0	50%	
J	Group	yes	85%	

Note. Numbers of clinicians who received training and passed the program are not provided to decrease the likelihood that the identities of agencies with clinicians who participated are discerned.

^†^At agencies with noncompleters, noncompletion rates ranged from 10–25%. Reasons for not completing include leaving the agency, personal circumstances, moved to another division of the agency that was not participating in training (e.g., intake department). No clinicians reported reasons for dropping out that were related to the training or consultation.
